# Psychometric properties of a prostate cancer radiation late toxicity questionnaire

**DOI:** 10.1186/1477-7525-5-29

**Published:** 2007-05-31

**Authors:** George Rodrigues, Glenn Bauman, Michael Lock, David D'Souza, Jeff Mahon

**Affiliations:** 1 Department of Oncology, University of Western Ontario, London, Ontario, Canada; 2 Department of Epidemiology and Biostatistics, University of Western Ontario, London, Ontario, Canada; 3 Department of Medicine, University of Western Ontario, London, Ontario, Canada

## Abstract

**Background:**

To construct a short prostate cancer radiation late toxicity (PCRT) questionnaire with health-related quality-of-life (HRQoL) domains.

**Methods:**

The PCRT was developed by item generation, questionnaire construction (n = 7 experts, n = 8 focus group patients), pilot testing (n = 37), item reduction (n = 100), reliability testing (n = 237), and validity testing (n = 274).

**Results:**

Reliability of the three item-reduced subscales demonstrated intraclass correlation coefficients (CC) of 0.811 (GU), 0.842 (GI), and 0.740 (sexual). Discriminant validity demonstrated Pearson CC of 0.449 (GU-GI), 0.200 (sexual-GU), and 0.09 (sexual-GI). Content validity correlations between PCRT-PCQoL were 0.35–0.78, PCRT-FACT-G^© ^were 0.19–0.39, and PCRT-SF-36^® ^were 0.03–0.34.

**Conclusion:**

We successfully generated a PCRT HRQoL questionnaire including subscales with very good psychometric properties.

## Background

The concept of therapeutic ratio is the relationship between tumor control and significant toxicities of treatment. The acute and long-term treatment-related morbidities associated with the various prostate cancer treatment options, such as radical prostatectomy (RP), external-beam radiation therapy (RT), permanent brachytherapy seed implant, temporary high-dose rate (HDR) brachytherapy implant, and hormonal manipulation, can be significant [1]. Therefore, the concept of therapeutic ratio is important in defining the trade-off that patients accept for cure/control vs. harm. Strategies incorporating radiation dose escalation (total dose and dose per fraction escalation) for improving local control require improvements in patient immobilization, prostate imaging/targeting, treatment delivery and verification. This increased level of technical sophistication is necessary in order to optimize the therapeutic ratio by adequately treating the tumor volume(s) of interest while respecting the tolerance of normal tissues such as rectum, bladder, penile bulb and the bilateral femoral heads [2]. Dose and dose-per-fraction escalation strategies to improve the prognosis of localized and locally advanced prostate cancer require a greater emphasis on the assessment and treatment of radiation-induced late effects. Better methods of documenting late rectal, bladder, and sexual toxicity are required to complement tumor control data such as the biochemical-free, disease-free, and overall survival endpoints to ensure the therapeutic ratio is being optimized through these treatment innovations.

Late rectal, bladder and sexual effects have been historically graded using toxicity scales such as the Radiation Therapy Oncology Group (RTOG)/European Organization for Research and Treatment of Cancer (EORTC) Late Radiation Morbidity Scoring Scheme or the Late Effects Normal Tissue Task Force – Subjective, Objective, Management, and Analytic (LENT-SOMA) scales [3-5]. These scales are usually easy to administer; however, they are limited in the type and complexity of the information captured. In addition, impact on health-related quality of life (HRQoL) of these side effects is not measured by these scales, (i.e. these scales do not measure the impact or bother to the patient of a specific symptom). Potentially, patients may have high symptom grade and low impact/bother or conversely can have low symptom grade and high impact/bother. Various HRQoL questionnaires (e.g. PCQoL, Expanded Prostate Index Composite [EPIC^©^]) have been constructed to assess HRQoL of patients with prostate cancer before, during and/or after various prostate cancer treatments (surgery, radiation, hormones, and brachytherapy) [6,7]. However, an easily administered questionnaire that can capture the late effects of RT has not been constructed [8,9].

The goal of this investigation was to construct a short prostate cancer radiation late-toxicity HRQoL instrument corresponding to relevant symptoms as identified by patients, radiation oncologists and the medical literature. This questionnaire was to be concise, easy to administer, and relate directly to the common late toxicities of prostate radiotherapy as assessed by patients, experts, and the medical literature of other existing HRQoL questionnaires and toxicity scales. Derivation of appropriate HRQoL subscales was to be performed though a process of item grouping and reduction. Various gastrointestinal (GI), genitourinary (GU) and sexual HRQoL subscales were to be generated and subsequently tested for ease of administration, reliability and validity.

## Methods

### Study questionnaires

#### Prostate Cancer Radiation Late Toxicity (PCRT) Questionnaire

The development of the PCRT questionnaire was the central focus of this research and the final questionnaire is included in Appendix 1 (see Additional file 1). The final PCRT is a 29-item instrument evaluating symptoms relating to the late effects of RT after treatment for prostate cancer. This study was designed to test 3 toxicity/QOL question subsets for the PCRT: GI Symptoms/Bother (Questions 1–12), GU Symptoms/Bother (Questions 13–23), and Sexual (Questions 24–28).

#### Prostate Cancer Quality of Life (PC-QoL) Questionnaire

The version one PC-QoL is a 52-item self-administered, prostate cancer health-related quality-of-life instrument relating to QOL issues for prostate cancer patients treated with a variety of local therapies (brachytherapy, RP, and external-beam RT)[6]. This questionnaire has ten HRQoL scales that can be self-administered in multiple settings (i.e. research vs. clinical). The subscales incorporated within the PC-QoL include: Urinary Function/Role Activity Limitations/Bother, Sexual Function/Role Activity Limitations/Bother, Bowel Function/Role Activity Limitations/Bother, and Cancer Worry.

#### Functional Assessment Cancer Therapy-General (FACT-G^©^)

The version four FACT-G^© ^is a 27-item general cancer health-related quality-of-life instrument with 5 QOL subscales which can be used in conjunction with modular instruments such as the FACT-P^©^[10]. Further refinements to the FACT-G^© ^questionnaire have resulted in the current 27-item version four questionnaire with the following five subscales: Physical Well-Being, Social-Family Well-Being, Emotional Well-Being, Functional Well-Being, and Overall Well-Being

#### Medical Outcomes Study Short Form – 36 (SF-36^®^)

The version one SF-36^® ^is a 36-item (short form health survey) general HRQoL with 8 subscales [11-13]. The SF-36^® ^was found to be reliable and valid in a variety of clinical/research settings and patient populations, both in the MOS study and in subsequent investigations. The eight health domains that the SF-36^® ^measures are: Health Perception, Physical Functioning, Role Limits in Physical Functioning, Role Limits in Emotional Health, Social Functioning, Mental Health, Pain, Energy and Fatigue.

#### Patient feedback/ease of use form

Patients' understanding of the PCRT, the ease of use form, and any other suggestions were obtained by having all patients complete a short questionnaire assessing the following domains: easy to understand questions, question format, adequate response range, and additional comments.

#### Study population

Five groups of patients were studied for the various stages of assessment of the PCRT questionnaire (1. questionnaire construction focus group (n = 8), 2. questionnaire pilot study – patient feedback (n = 37), 3. item reduction (n = 100), 4. reliability testing (n = 237), and 5. validity testing (n = 271). A total of 479 patients were approached for various aspects of the study with 379 (79%) patients agreeing to participate. All five populations used the same inclusion and exclusion criteria as described below.

The study population consisted of male patients of any age who had previously undergone radical external beam RT or radical brachytherapy for prostate cancer at the London Regional Cancer Program (LRCP). Patients with any T stage, PSA, and Gleason score were eligible for the study. Participants were selected from the GU follow-up clinics provided they met all required inclusion criteria. The study involved those individuals who had completed radiation at least one full year prior to their follow-up appointment. Patients were excluded from the study if they have had previous surgery (i.e. RP or orchidectomy), chemotherapy, or any clinical or radiological evidence of metastases. Patients with PSA failure, previous or current hormonal therapy, or TURP procedures were allowed in the study. Patients must have been able to complete questionnaires in English.

#### Construction of PCRT questionnaire

This goal of this study was to construct a brief < 30-item questionnaire assessing late toxicities relating to prostate cancer radiotherapy. After a systematic review and assessment of the world literature (PUBMED abstracts of prostate cancer HRQoL questionnaires and their respective reference lists as well as published prostate cancer HRQoL review article reference lists), a list of potentially relevant items was obtained. Questionnaire items were chosen and constructed on a 5-point Likert scale. A total of twenty-nine questions were constructed assessing the GU, GI, and sexual late toxicities in terms of symptom severity and its associated bother to the patient. Questionnaire items were constructed with wording consistent with an eighth grade level.

Consultation with several LRCP staff radiation and medical oncologists at the LRCP (n = 7) was performed to evaluate the appropriateness of the initial questionnaire, and its face validity. After further editing, the questionnaire was assessed and completed by 8 prostate cancer patients attending a local prostate cancer support group for feedback on content and wording. No demographic information was available in this cohort due to privacy concerns. All necessary final revisions were made at this point in order to improve the questionnaire prior to general implementation.

#### Pilot testing of PCRT questionnaire

A convenience sample of 37 patients was obtained at the LRCP consistent with the study inclusion/exclusion criteria. Patient demographic information was collected and each patient filled in a patient feedback/ease of use questionnaire in conjunction with a PCRT questionnaire. The PCRT questionnaire was administered to this cohort of patients in order to determine patient's perceptions of whether the content and format of the questionnaires were easy to understand. In addition, subjects were asked to comment on whether any additional items should be included.

#### Item reduction of PCRT questionnaire

A PCRT questionnaire and a self-addressed, self-stamped envelope were provided to eligible patients. A sample of 100 patients was planned for this phase of the study. Each participant was given one day to complete the PCRT instrument and then returned the package to the study investigators. Item reduction was performed by the deletion of low variability items. If > 90% of responses were identical for all symptom and bother items in a specific domain, all questions were deleted from the appropriate scales. For example, if both the diarrhea "symptom" and "bother" question were both found to have low heterogeneity of response, and then both questions were deleted from their appropriate scales. If only one of the questions was found to have low variability, neither question was deleted at this stage of the analysis; however, the potential impact of their deletion was assessed by Cronbach sensitivity analysis [14].

Cronbach coefficient sensitivity analysis for deleted items was used to determine if any of the remaining items should be deleted. Cronbach alpha values of greater than or equal to 0.7 are considered to demonstrate good internal subscale consistency. Values of 0.5 to 0.7 are considered to be acceptable with values under 0.5 considered to be an unacceptable level of internal consistency. Therefore, for scales with less than 0.7 Cronbach alpha statistics, sensitivity analysis was used to determine the optimal number of items in the scale to maximize the alpha coefficient. No imputation of missing values was performed; therefore, only subscales with complete data were used for Cronbach's analysis.

#### Reliability testing of PCRT questionnaire

The test-retest reliability of the PCRT questionnaire was determined by comparing the consistency of answers between a first self-administration of the PCRT questionnaire and a second self-administration two weeks later on eligible study patients. Two weeks after the initial questionnaire was completed, a reminder phone call was placed to all patients prompting them to complete the second questionnaire and to then send it back to LRCP. Patient demographic statistics and questionnaire response descriptive statistics were both calculated. Raw subscale scores were used with no linear transformation for this analysis. Intraclass correlation coefficients (one way, two-way fixed effects model, and two-way random effects model) and 95% confidence intervals for all PCRT subscales were calculated to assess the reliability of the questionnaire. No imputation of missing values was performed; therefore, only subscales with complete data were used for initial intraclass correlation coefficient analysis. A second analysis including subscale scores with ≥ 50% data completion was also performed. Intraclass correlation coefficients of > 0.7 were considered to be indicative of an excellent reliability correlation. Intraclass correlations of 0.4 – 0.7 were considered to be acceptable.

#### Validity testing of PCRT questionnaire

Individuals fitting the study parameters were provided with questionnaire validation packages. The package consisted of an envelope with a set of four questionnaires (PCRT, PC-QoL, FACT-G^©^, and SF-36^®^) given in random order (block randomized in groups of 24 = 4 × 3 × 2 × 1) and a self-addressed stamped envelope. The participant was given one day to complete the questions and then was to return the package to the LRCP. The data collected from these questionnaires was used to assess the validity of the PCRT questionnaire. Subscale scores were calculated and subjected to a linear transformation to a 0 (low HRQoL, high symptoms) to 100 (high HRQoL, low symptoms).

To assess discriminant validity, Pearson correlation coefficients were calculated among all the subscales of the PCRT questionnaire. No imputation of missing values was performed; therefore, only subscales with complete data were used for Pearson correlation coefficient analysis. To assess construct validity, Pearson correlation coefficients were calculated between all subscales of the PCRT questionnaire and the PC-QoL; PCRT and FACT-G^©^; PCRT and SF-36^®^. To further assess construct validity, a comparison of PCRT scores between brachytherapy and external-beam patients was performed using Student's t-test (as brachytherapy and external-beam RT patients would be expected to have different toxicity profiles).

#### Sample size considerations

The sample size for the initial pilot study of 37 patients was based on a convenience sample. The sample size calculation for the internal consistency evaluation of 100 patients (37 pilot + 63 additional patients) was based on the confidence interval formula [N = (z_α/2 _/CI)^2^+3, where N = sample size, z_α/2 _= 1.96 for 95% confidence interval CI is the correlation coefficient confidence interval]. Therefore, for a confidence interval of 0.2, the required sample size was calculated to be 99 patients.

The anticipated sample size for the combined reliability and validation study will be approximately 272 completed questionnaire sets in order to detect statistically significant correlations of 0.30 or higher (0.00 – null hypothesis). A sample size of 191 individuals was calculated; however, the sample size was increased by 50% in order to take into account potential missing data from questionnaire non-compliance. This calculation has also taken multiple statistical testing into account with a Bonferroni correction calculation (alpha = 0.05/140 = 0.00035). All statistics were performed on the SAS/STAT^® ^(SAS Institute^®^, Cary NC, USA) system.

## Results

### Construction of PCRT questionnaire

After a medical literature review and discussion with expert GU oncologists an initial questionnaire of twenty-nine 5-point Likert scale items was created. The final list of items included questions encompassing the GU (frequency/nocturia, dysuria, hematuria, and incontinence), GI (hematochezia, diarrhea, pelvic pain, bowel control, and tenesmus), and sexual (impotency, libido, and interest) domains. Questionnaire items either related to the quantitative degree of impairment of the particular "symptom" or to the qualitative "bother" experienced by patients for a particular symptom.

All 8 patients approached to fill out an initial questionnaire during the London Prostate Cancer Support Group meeting completed questionnaires. Most questionnaire items demonstrated some variability in response other than hematuria (symptoms/bother) and incontinence (symptom only). All other questionnaire items had significantly greater variability in response. A questionnaire was constructed after all patient, expert, and medical literature consultation was complete (Appendix 1 [see Additional file 1]).

### Pilot testing of PCRT questionnaire

Of 40 patients who were eligible and who agreed to participate in the study, 37 (93%) completed a PCRT questionnaire and patient feedback form. Mean patient age was 74 years (range 61 – 82 years). Pre-treatment parameters were as follows. Mean PSA was 14.9 ng/ml (range 2.0 – 68.0). T staging was T1 (10 patients, 27%), T2 (21 patients, 57%), and T3 (6 patients, 16%). Median Gleason grade was 6/10 with 9 (24%) patients with grade 2–5, 13 (35%) patients with grade 6, 10 (27%) patients with grade 7, and 5 (14%) patients with grade 8–10. Median prostate dose to the prostate was 66 Gray (Gy, range 63 – 76Gy) with 10/37 (27%) patients receiving pelvic RT to regional nodes and prostate (median dose 46 Gy, range 44 – 50 Gy). No patients were treated with brachytherapy.

Only 14 (1.3%) among 1073 (37 questionnaires × 29 questions per questionnaire) possible responses were missing. All missing items were related to sexual questionnaire items. Thirty-three (89%) of thirty-seven pilot study patients filled out the patient feedback form after completion of the PCRT questionnaire. All patients reported that the questionnaire items and format were easy to understand (feedback form question 1,3). Of the 33 respondents, 31 (94%) patients found the questionnaire comprehensive, with 1 (3%) patient not responding, and 1 (3%) patient recommending the addition of a question assessing fatigue. No pilot study patients had any further comments or potential changes to recommend.

### Item reduction of PCRT questionnaire

One hundred and twenty-six patients were consented for completion of PCRT questionnaires for item reduction with 100 (79%) patients completing the PCRT. Of patients filling out the questionnaire, mean patient age of respondents was 75 years (range 61 – 82 years). Pre-treatment parameters were as follows. Mean PSA was 15.1 ng/ml (range 2.0 – 68.0). Prostate cancer T staging was T1 (20 patients, 20%), T2 (59 patients, 59%), T3 (18 patients, 18%) and Tx (3 patients, 3%). Median Gleason grade was 6/10 with 49 (49%) patients with grade 2–6, 34 (34%) patients with grade 7, and 16 (16%) patients with grade 8–10. Median prostate dose was 69.4 Gy (range 52.5 – 76.0 Gy) with 27/100 (27%) patients receiving pelvic RT to regional nodes and prostate (median dose 46 Gy, range 44 – 50.4 Gy). No patients were treated with brachytherapy. No demographic information was collected on non-responders due to privacy/ethics concerns.

Of 100 patients who filled out questionnaires, 77 (77%) had no missing items. Eleven patients (11%) had only one missing item and a further 11 (11%) patients had 2–6 missing items. One patient (1%) has 18 missing items. Of a possible 2900 (100 questionnaires × 29 questions per questionnaire) responses, 71 (2.4%) were missing. Eighteen of 1200 (1.5%) GI responses, 10 of 1100 (0.9%) GU, and 43 of 600 (7.2%) sexual responses were missing.

In terms of question response extremes; frequency (q13, n = 2), nocturia (q14, n = 4), impotency (q24, n = 1), libido (q26, n = 2), and sexual contentment (q28, n = 4) had less than ten percent #1 (least affected) responses. All sexual questions (q24-q28) had greater than ten percent #5 (most affected) responses. Conversely, none of the other questions (q1-q23) had greater than five percent #5 responses. The following questions were found to have limited heterogeneity (> 90% in one response item): dysuria symptoms and bother (q16-q18), hematuria symptoms and bother (q19-q20), and incontinence II symptom and bother (q22-q23). The first incontinence symptom question (q21) had sufficient heterogeneity of response (#1 – 74%, #2 – 15%, #3 – 2%, #4 – 6%, #5 – 1%) to maintain the entire incontinence question set in the GU subscale. Therefore, the dysuria and hematuria questions sets were deleted from further consideration within the GU subscales.

Cronbach coefficient analysis using three subscales demonstrated raw coefficients for GI (0.859), GU (0.529), and sexual (0.700) subscales (Table 1). No significant improvements in coefficients were found with the deletion of any questionnaire item from their respective subscale except for libido symptom (q26, 0.700 to 0.756). However, removal of the corresponding bother question would decrease the Cronbach's coefficient (0.700 to 0.574). Therefore, no further questions were deleted from their respective subscales. Splitting the GI and GU domains into separate symptom and bother subscales diminished the Cronbach's coefficients in all cases: GI symptoms (0.859 to 0.729), GI bother (0.859 to 0.777), GU symptoms (0.529 to 0.358), and GU bother (0.529 to 0.190). Therefore, three subscales were used for further reliability and validity testing.

**Table 1:** Item reduction analysis – Cronbach coefficient alpha analysis (3 subscales)

**Deleted Questionnaire Item**	**GI (n = 93)**	**GU (n = 97)**	**Sexual (n = 85)**
q1	0.848		
q2	0.851		
q3	0.853		
q4	0.853		
q5	0.841		
q6	0.851		
q7	0.848		
q8	0.848		
q9	0.851		
q10	0.843		
q11	0.848		
q12	0.831		
q13		0.481	
q14		0.502	
q15		0.458	
q21		0.268	
q22		0.497	
q23		0.528	
q24			0.699
q25			0.588
q26			0.756
q27			0.574
q28			0.560
Raw Cronbach Coefficient alpha	0.859	0.529	0.700

A Cronbach alpha sensitivity analysis of a four-item GU scale (all frequency/nocturia and incontinence I symptoms) versus a six-item scale (all frequency/nocturia and incontinence items) versus an 11-item scale (all potential GU items) was performed to identify the optimal number of scale items (Table 2). The maximum raw Cronbach coefficient was found in the scale including only questions that were previously identified as having acceptable heterogeneity of response (4-item GU scale). Additional items did not improve internal consistency to the GU scale (Cronbach alpha 0.545 (4-item) versus 0.529 (6-item) and 0.515 (11-item).

**Table 2:** Item reduction analysis – Cronbach sensitivity analysis for GU domain

**Deleted Question #**	**Domain**	**GU 4-item**	**GU 6-item**	**GU 11-item**
q13	Frequency (s)	0.485	0.481	0.470
q14	Nocturia (s)	0.518	0.502	0.488
q15	Frequency/Nocturia (b)	0.328	0.364	0.381
q16	Dysuria I (s)			0.518
q17	Dysuria II (s)			0.518
q18	Dysuria (b)			0.504
q19	Hematuria (s)			0.511
q20	Hematuria (b)			0.520
q21	Incontinence I (s)	0.533	0.489	0.463
q22	Incontinence II (s)		0.497	0.471
q23	Incontinence (b)		0.529	0.504
Raw Cronbach Coefficient Alpha		0.545	0.529	0.515

### Reliability testing of PCRT questionnaire

Three hundred and forty-two patients were approached regarding the reliability study with 274 (80%) consenting to participate in the study. Two hundred and seventy-one patients filled out the PCRT test questionnaire, with 237 (87%) patients filling out the subsequent retest questionnaire. Of patients consenting to the study, mean patient age of respondents was 73.8 years (range 56 – 88 years). Pretreatment parameters were as follows. Mean PSA was 13.3 ng/ml (range 2.0 – 144.0). Prostate cancer T staging was T1 (78 patients, 28.5%), T2 (142 patients, 51.8%), T3 (40 patients, 14.6%) and Tx (14 patients, 5.1%). Median Gleason score was 6/10 with 142 (51.8%) patients with grade 2–6, 81 (29.6%) patients with score 7, and 38 (13.9%) patients with score 8–10. Median prostate dose was 70.0 Gy (range 50.0 – 78.0 Gy) with 10/232 (4.3%) patients receiving pelvic RT (median dose 46 Gy, range 44 – 50.4 Gy). Thirty-eight patients were treated with brachytherapy. No demographic information was collected on non-responders due to privacy/ethics concerns.

Calculation of the one-way and two-way intraclass correlation coefficients (ICC) was performed (Table 3). Estimates of GI, GU, and sexual ICC for the no-missing test-retest reliability condition were 0.842, 0.811, and 0.740, respectively. The corresponding values for ICC when less than or equal to 50% missing data is allowed are 0.784, 0.779, and 0.729. Therefore, in general test-retest reliability was found to be very good in both the complete data and partially complete data settings.

**Table 4:** Validity analysis – Pearson correlation coefficient content validity analysis (PCQoL)

**PCQoL Domain**	**GI PCRT**	**Sexual PCRT**	**GU PCRT**
	0.205	0.24	0.54
Urinary Function	**p = 0.002**	**p = 0.0004**	**p < 0.0001**
	n = 216	n = 205	n = 243
	0.08	0.280	0.35
Urinary Limitations	p = 0.224	**p < 0.0001**	**p < 0.0001**
	n = 218	n = 207	n = 244
	0.29	0.280	0.64
Urinary Bother	**p < 0.0001**	**p < 0.0001**	**p < 0.0001**
	n = 215	n = 205	n = 240
	0.04	0.420	0.09
Sexual Function	p = 0.531	**p < 0.0001**	**p = 0.018**
	n = 207	n = 207	n = 232
	0.18	0.480	0.18
Sexual Limitations	**p = 0.009**	**p < 0.0001**	**p = 0.005**
	n = 204	n = 205	n = 227
	0.25	0.660	0.15
Sexual Bother	**p = 0.0004**	**p < 0.0001**	**p = 0.025**
	n = 203	n = 205	n = 225
	0.78	0.20	0.45
Bowel Function	**p < 0.0001**	**p = 0.004**	**p < 0.0001**
	n = 219	n = 205	n = 244
	0.61	0.27	0.38
Bowel Limitations	**p < 0.0001**	**p < 0.0001**	**p < 0.0001**
	n = 219	n = 208	n = -246
	0.73	0.25	0.46
Bowel Bother	**p < 0.0001**	**p = 0.0003**	**p < 0.0001**
	n = 219	n = 206	n = 245
	0.16	0.12	0.16
Cancer Worry	**p = 0.016**	p = 0.074	**p = 0.011**
	n = 220	n = 206	n = 246

### Validity testing of PCRT questionnaire

Three hundred and forty-two patients were approached regarding the study at LRCP of which 274 (80%) agreed to participate. The PCRT test questionnaire was filled out by 271 individuals, and the PC-QoL (n = 272), FACT-G^© ^(n = 271), SF-36^® ^(n = 272) questionnaires were filled out by similar numbers. Patient demographics and radiation statistics were identical to the reliability testing cohort as all patients that consented to the reliability testing study also completed the validity questionnaire set.

Mean PCRT subscale transformed scores were 89.6 (GI), 66.9 (GU), and 42.0 (sexual). Mean PCQoL domain scores ranged from a low of 20.1 (sexual function) to a high of 94.5 (bowel limitations) with a possible range of 0 (severely affected) to 100 (not affected). Mean FACT-G^© ^domain scores ranged from 20.8 (emotional domain) to 24.6 (physical domain) with a range of 0 (severely affected) to 28 (not affected). Mean SF-36^® ^domain scores ranged from 56.1 (role physical) to 85.6 (social functioning) with a range of 0 (severely affected) to 100 (not affected).

The highest intra-PCRT domain Pearson correlation exists with GI-GU at 0.449. Lower correlations existed between the sexual and GU (0.200) and GI (0.098) domains. Pearson correlation between the PCRT and PCQoL domains are presented in table 4. Significant correlations between analogous domains were demonstrated. GI PCRT correlations with bowel-related PCQoL domains ranged from 0.61 to 0.78. GU PCRT correlations with urinary-related PCQoL domains ranged from 0.35 to 0.64. Sexual PCRT correlations with related PCQoL domains ranged from 0.42 to 0.66. Correlation between the GI PCRT and FACT-G^© ^domains ranged from 0.19 to 0.34 (Table 5). GU PCRT correlations with FACT-G^© ^domains ranged from 0.20 to 0.39. Correlation between the PCRT sexual domain and the FACT-G^© ^domains ranged from 0.21 to 0.35. Correlation between the GI PCRT and SF-36^® ^domains ranged from 0.03 to 0.27 (Table 6). GU PCRT correlations with SF-36^® ^domains ranged from 0.16 to 0.34. Correlation between the PCRT sexual domain and the SF-36^® ^domains ranged from 0.14 to 0.55.

**Table 5:** Validity analysis – Pearson coefficient content validity analysis (FACT-G^©^)

**FACT-G^© ^Domain**	**GI PCRT**	**Sexual PCRT**	**GU PCRT**
Physical	0.34	0.34	0.39
	**p < 0.0001**	**p < 0.0001**	**p < 0.0001**
	n = 220	n = 205	n = 243
Social/Family	0.19	0.21	0.20
	**p = 0.005**	**p = 0.003**	**p = 0.002**
	n = 219	n = 203	n = 242
Emotional	0.20	0.22	0.24
	**p = 0.002**	**p = 0.002**	**p = 0.0002**
	n = 219	n = 206	n = 244
Functional	0.21	0.27	0.29
	**p = 0.0001**	**p < 0.0001**	**p < 0.0001**
	n = 222	n = 208	n = 247
Total	0.32	0.35	0.31
	**p < 0.0001**	**p < 0.0001**	**p < 0.0001**
	n = 216	n = 201	n = 239

**Table 6:** Validity analysis – Pearson correlation coefficient content analysis (SF-36^®^)

**SF-36^® ^Domain**	**GI PCRT**	**Sexual PCRT**	**GU PCRT**
	0.12	0.55	0.16
Physical Functioning	p = 0.06	**p = 0.027**	**p = 0.013**
	n = 220	n = 205	n = 244
	0.18	0.22	0.20
Role Physical	**p = 0.007**	**p = 0.002**	**p = 0.0018**
	n = 219	n = 207	n = 242
	0.21	0.17	0.31
Bodily Pain	**p = 0.002**	**p = 0.014**	**p < 0.0001**
	n = 219	n = 207	n = 245
	0.26	0.14	0.23
General Health	**p < 0.0001**	**p = 0.05**	**p = 0.0003**
	n = 220	n = 206	n = 245
	0.25	0.26	0.26
Validity	**p = 0.0002**	**p = 0.0002**	**p < 0.0001**
	n = 221	n = 209	n = 248
	0.27	0.24	0.34
Social Functioning	**p < 0.0001**	**p = 0.0004**	**p < 0.0001**
	n = 219	n = 206	n = 246
	0.03	0.21	0.19
Role Emotional	p = 0.63	**p = 0.0004**	**p = 0.004**
	n = 213	n = 198	n = 237
	0.26	0.48	0.22
Mental Health	**p = 0.0002**	**p = 0.01**	**p = 0.0004**
	n = 221	n = 209	n = 248

Higher mean domain scores were found in the brachytherapy cohort when compared to the external-beam radiation population. Mean difference scores of 4.13, 3.50, and 3.63 were found for the GI, sexual, and GU domains respectively. Only the GI domain was found to have a statistically significant difference in score with a p-value of 0.02.

## Discussion

As with any symptom or side effect of treatment, there are quantitative and qualitative aspects to the potential impairment. The level of impairment (quantitative symptom scale) to the patient can range from no impairment to severe impairment. The potential impact of the symptom (qualitative bother scale) to the patient can also vary from no bother to severe bother. The PCRT questionnaire was constructed to capture both symptom and bother concepts for each of the late sexual, GI, and GU impairments represented in the questionnaire. The questionnaire was constructed so that if a patient responded that they did not experience a specific symptom, they would skip the corresponding "bother" question because it was irrelevant to that patient. A five-point scale was used in each of the questionnaire items in order to balance sufficient discriminative symptom and bother response levels with easy administration and reporting properties. In addition, cancer clinicians are familiar with the concept of 5-point toxicity scales ranging from low to high impairment. Commonly used instruments include the NCICTC, RTOG Radiation Toxicity Criteria, and the Eastern Cooperative Oncology Group (ECOG) Performance Status scale.

During the initial questionnaire construction process, eight prostate cancer patients conforming to the study population filled out questionnaires. The primary role of the initial group of eight patients was to give initial questionnaire feedback to the investigator for improvements in wording and content. Due to the limited number of initial patients looking at the questionnaire, no formal conclusions regarding comprehensiveness and ease of use could be made. No changes in the questionnaire items were made prior to further pilot testing and item reduction analysis. Patients were asked to assess the various symptoms and corresponding bother item over the last four weeks prior to filling out the questionnaire. This was done in order to reduce the impact of variability of filling out the questionnaire on a non-representative day. Because most toxicities occur in the order of several months to many years after RT, the four week time period was felt to be appropriate. In addition, follow-up visits after prostate cancer radiation occur usually in three to twelve month intervals.

Response rates for both the PCRT questionnaire and the corresponding patient feedback form were high at 93% and 89%, respectively. The pilot study also allowed an assessment of missing items. The missing item rate was found to be low at 1.3% of all possible responses. All missing responses dealt with the sexual domain of the PCRT, likely a reflection of the personal nature of the questions. However, over 90% of all sexual domain questions were answered by the respondents, likely due to the general non-explicit nature of the questions and that only six questions of the 29 dealt with this issue. In addition, the pilot cohort could be considered to be a select group of motivated patients, which would lead to generally higher questionnaire and questionnaire item response rates. All pilot study patients reported that the questionnaire content and format were easy to understand. The response rate for the item reduction cohort PCRT questionnaire was 79% compared to 93% in the initial pilot cohort. This potentially reflects a less motivated group of patients and/or less intensive monitoring during data collection given a larger study population as compared to the pilot cohort. The assessment of missing items demonstrated a low overall missing item response rate of 2.4% with the majority of missing items coming from the sexual question set (7.2%) versus the GI (1.5%) or GU (0.9%) question sets. Therefore, the observation of a disproportionate level of missing data with the sexual question set was confirmed in the larger item reduction cohort.

Ninety-three percent of patients "never" experienced dysuria and 97% "never" had hematuria. Dysuria is a common acute side-effect of radiation treatment which usually subsides after completion of therapy. Gross hematuria (visible to the naked eye) is an uncommon late side-effect of RT. The finding of hematuria and dysuria as low variability items in a late toxicity questionnaire was an expected finding. In general, clinically relevant and significant late toxicities such as hematuria, which occur infrequently, may be better assessed in a late toxicity scale (RTOG, LENT-SOMA) as opposed to a HRQoL instrument. In terms of the integration of these items into the GU HRQoL scale of the PCRT it was felt it would be inappropriate given the lack of discriminative ability of these two question sets within the population of patients being studied.

For the subscale analysis, the GI scale had very good internal consistency (0.859), the sexual scale demonstrated good consistency (0.700), and the GU scale demonstrated fair consistency (0.529). An assessment of the various options for a 4-, 6-, and 12-item GU scale demonstrated a slight advantage to the shorter 4-item scale on Cronbach sensitivity analysis. The shorter 4-item scale corresponds to the 4 items that had significant heterogeneity of response: Frequency symptoms, Nocturia symptoms, Frequency/nocturia bother, and Incontinence (frequency of leaking urine) symptoms. The decreased internal consistency of the GU scale may potentially be a result of diminished variability compared to the sexual and GI scales in this study population (majority treated with external-beam RT with minority brachytherapy). In addition, further refinement of the GU questionnaire items may improve internal consistency. Potentially, using more than five question responses for the GU questionnaire items may increase the variability of response and the discriminative ability of the scale.

Further reliability testing (test-retest) and validity analyses were performed to evaluate the characteristics of the 4-item GU scale in order to make a final determination of its appropriateness as a HRQoL scale. Therefore, the final PCRT questionnaire contains 29-items with GI (12-item), GU (4-item), and sexual (5-item) subdomains, and 7 individual dysuria, hematuria, and incontinence pad/diaper symptom and bother items. In addition, q29 separately assesses level of sexual activity but is not included in the sexual score. The reliability test-retest analysis had an excellent overall 80% response rate that will likely not cause any issues regarding selection bias and the generalisability of the questionnaire. All reliability intra-class correlations were greater than or equal to 0.7 for all domains either with no missing data or < 50% missing data. Slightly higher reliability coefficients were seen in the analysis of patients with no missing data due to a highly consistent dataset. However, the differences in reliability coefficient due to missing data were small and in the range of 0.011 to 0.059. The one-way intraclass correlation coefficient is likely the best estimate for reliability given the random nature of patients fully completing the domain of interest. Therefore, the PCRT domains demonstrated stable and consistent mean and median scores in all domains with reasonable overall and individual subscale response rates. Reliability, as assessed by the test-retest intraclass correlation coefficient, was acceptable for all three subscales. Therefore, validation proceeded for all three PCRT subscales.

The overall response rate for all patients approached regarding the validation study was acceptable at 80%. Of the 274 patients who completed the four questionnaire validation sets, over 99% of the questionnaires were completed (PCRT n = 271, PCQoL n = 272, FACT-G^© ^n = 271, and SF-36^® ^n = 272). This population demonstrated different levels of HRQoL for different domains of the PCRT questionnaire. It appears that these patients have overall good GI, intermediate GU, and poor sexual functioning as measured by the PCRT instrument. Poor sexual function scores were also seen in the PCQoL questionnaire. In addition, this study population demonstrated low average vitality and physical scores on the SF-36^® ^questionnaire. The FACT-G^© ^mean scores were relatively homogeneous with no domain being more affected than the others. The PCRT questionnaire demonstrated discriminative validity in terms of the fact that there was some low-level correlation between all three measures ranging from 0.098 to 0.449. Therefore, the three subscales measure different but somewhat related (i.e. relating to radiation toxicities) domains. Further content validity was demonstrated by the progressive lower correlation between the subscales of the PCRT with the prostate cancer questionnaire (PCQoL), the general cancer questionnaire (FACT-G^©^), and a general health questionnaire (SF-36^®^).

The limitations of the study include the following: the PCRT is validated in external-beam RT and brachytherapy populations only (surgery, chemotherapy populations not studied), the PCRT is only validated for the 4 week time frame with the PCRT not designed for the acute phase of radiation (either concurrently with RT and/or < 6 months after RT complete), the PRCT subscales have not been cross-validated against existing late toxicity scales, or other toxicity risk factors such as DVH parameters, and responsiveness of PCRT subscales over time has not been determined. In addition, limited psychometric testing in the brachytherapy population may have excluded the dysuria question pair (symptom and bother) from the final GU subscales.

Future work in the development of the PCRT questionnaire can include an assessment of other populations that receive external-beam RT for prostate cancer would generate additional information regarding the PCRT questionnaire. Unstudied populations include patients who have had previous TURP or RP (adjuvant or salvage setting) and patients who may receive concurrent chemotherapy with radiotherapy. These populations may potentially express greater levels of late toxicity due to the multimodality nature of the treatments given. Study of subpopulations of RT patients (nodal RT vs. prostate alone, moderately high vs. high dose RT) may also be performed. An acute version of the questionnaire has been developed and will be tested in an appropriate patient population.

Integration of the PCRT questionnaire into clinical trials assessing novel radiation techniques, dose escalation and dose per fraction escalation can be performed. Assessment of new normal-tissue sparing technologies such as IMRT and helical tomotherapy require validated toxicity and HRQoL instruments to assess potential improvements. Potential randomized phase II or III studies could use the PCRT as either a primary (phase I/II safety assessment) or secondary (phase III superiority or equivalence trial) endpoint. Concurrent chemotherapy with agents such as taxanes may also become increasingly the focus of clinical trials. Assessment of late effects will be necessary to confirm the safety of the combined management should efficacy be detected. Correlation with existing late toxicity scales and dosimetric/DVH parameters could potentially be another avenue for investigation. Routine intermittent administration of this questionnaire in the setting of post-radiation therapy follow-up could serve as a screening questionnaire to detect negative quality-of-life effects due to the late toxicities of prostate radiation therapy. Identified individuals with large changes in late toxicity quality of life scores can be subsequently subjected to appropriate diagnostic, therapeutic, and educational programs to mitigate these negative quality-of-life effects.

## Conclusion

We successfully generated a PCRT HRQoL questionnaire including genitourinary, gastrointestinal, and sexual subscales. This research has created a psychometrically reliable and valid short questionnaire specifically assessing the HRQoL related to the late effects of prostate radiation therapy. This instrument should have important future applications in the long-term clinical follow-up/triage of radiation late effects. In addition, various clinical trial/research opportunities exist in association with such a targeted questionnaire.

## Competing interests

The author(s) declare that they have no competing interests.

## Authors' contributions

GR was the principal investigator of this work in terms of protocol initiation, data analysis, interpretation, and manuscript preparation. GB was a member of GR Master's Thesis committee and supervised GR's conduct of this research. ML and DD are Radiation Oncologists would participated in the initial project meetings of the study, enrolled patients on the study, and reviewed and edited the manuscript prior to submission. JM was GR's Master's Thesis supervisor and supervised all aspects of this project including final manuscript preparation.

**Table 3:** Reliability Analysis – Intraclass Correlation Coefficient Test-Retest Analysis

Domain	ICC	No Missing Data (95% CI)	≤ 50% Missing Data (95% CI)
GI	one way	0.842 (0.792–0.881)	0.784 (0.728–0.829)
	two way (random)	0.842 (0.281–0.881)	0.784 (0.252–0.828)
	two way (fixed)	0.843 (0.794–0.881)	0.784 (0.728–0.825)
GU	one way	0.812 (0.760–0.853)	0.779 (0.723–0.825)
	two way (random)	0.811 (0.767–0.852)	0.779 (0.789–0.824)
	two way (fixed)	0.811 (0.759–0.853)	0.778 (0.723–0.824)
Sexual	one way	0.740 (0.664–0.801)	0.729 (0.660–0.787)
	two way (random)	0.740 (0.294–0.801)	0.729 (0.263–0.787)
	two way (fixed)	0.740 (0.663–0.801)	0.729 (0.659–0.787)

Random = random effects model; Fixed = fixed effects model.

## Supplementary Material

Additional file 1 Rodrigues Appendix. doc. APPENDIX 1: The Prostate Cancer Late Radiation Toxicity Questionnaire (PCRT). Click here for file
