# Supplementary material for: Psychometric properties of a prostate cancer radiation late toxicity questionnaire
Source: Health Qual Life Outcomes. 2007 May 31;5:29. doi: 10.1186/1477-7525-5-29 (PMC1894787; doi:10.1186/1477-7525-5-29)
Supplement: Additional file 1 — Rodrigues Appendix. doc. APPENDIX 1: The Prostate Cancer Late Radiation Toxicity Questionnaire (PCRT). [file 1477-7525-5-29-S1.doc]

APPENDIX 1: The Prostate Cancer Radiation Late Toxicity Questionnaire (PCRT)

The following self-reporting questionnaire looks at the long term side effects you may have experienced from your prostate cancer radiation therapy. The questions ask about your bowel and urinary habits, and your sexual functioning. While these questions are very personal in nature, answering them as honestly as possible is important to us in choosing the best possible follow-up care for you.

Please circle your answer.

The following questions deal with your daily bowel habits. Some questions are very personal in nature and you may leave them blank if they make you uncomfortable. Your responses will be kept confidential.

Please circle the answer that best describes your situation.

1. In the past 4 weeks, how often have you had blood in your bowel movements?

# Never

Sometimes

Frequently

Most of the time

All or almost all of the time

**IF NEVER, SKIP TO QUESTION 4**

1. On average, over the past 4 weeks, how much blood have you had in your bowel movements?

# None

Slight tinge

Light bleeding

Medium bleeding

Heavy bleeding

1. On average, over the past 4 weeks, how much upset or disruption in your daily activities has the blood in your bowel movements caused you?

No upset or disruption

Very little upset or disruption

# Small upset or disruption

Moderate upset or disruption

Severe upset or disruption

1. On average, over the past 4 weeks, how many loose or liquid bowel movements per day did you have?

None (or constipated)

Less than one loose or liquid bowel movement per day

One loose or liquid bowel movement per day

Between two and four loose or liquid bowel movements per day

# Five or more loose or liquid bowel movements per day

###### IF NONE, SKIP TO QUESTION 6

1. On average, over the past 4 weeks, how much upset or disruption in your daily activities have the loose or liquid bowel movements caused you?

No upset or disruption

# Very little upset or disruption

Small upset or disruption

Moderate upset or disruption

Severe upset or disruption

1. Over the past 4 weeks, how often have you experienced pelvic pain or cramping?

# Never

Sometimes

Frequently

Most of the time

Always or almost always

###### IF NEVER, SKIP TO QUESTION 9

7. On average, over the past 4 weeks, how severe has the pelvic pain or cramping been?

Not uncomfortable

Mildly uncomfortable

Somewhat uncomfortable

Moderately uncomfortable

Very uncomfortable

8. On average, over the past 4 weeks, how much upset or disruption in your daily activities has your pelvic pain or cramping caused you?

# No upset or disruption

Very little upset or disruption

Small upset or disruption

Moderate upset or disruption

Severe upset or disruption

9. On average, over the past 4 weeks, how often have you felt the urge to have a bowel movement and not had one?

Never

Rarely

Frequently

Most of the time

All or almost all of the time

**IF NEVER, SKIP TO QUESTION 11**

10. On average, over the past 4 weeks, how much upset or disruption in your daily activities has having the urge to have a bowel movement caused you?

No upset or disruption

Very little upset or disruption

Small upset or disruption

Moderate upset or disruption

Severe upset or disruption

11. On average, over the past 4 weeks, how much control have you had over your bowels?

Total control

Control most of the time

Some control

Very little control

No control

###### IF YOU HAVE HAD TOTAL CONTROL, SKIP TO QUESTION 13

12. On average, over the past 4 weeks, how much upset or disruption in your daily activities has your degree of bowel control caused you?

No upset or disruption

Very little upset or disruption

Small upset or disruption

# Moderate upset or disruption

# Severe upset or disruption

Urinary and Bladder Habits

The following questions deal with your daily urinary and bladder habits. Some questions are very personal in nature and you may leave them blank if they make you uncomfortable. Your responses will be kept confidential.

Please circle the answer that best describes your situation.

13. On average, over the past 4 weeks, how often did you urinate during the course of your day?

Two or fewer times

Between three and five times

Between six and eight times

# Between nine and twelve times

Thirteen times or more

14. On average over the past 4 weeks, how often did you have to get up in the night to go to the bathroom to urinate?

Never

Occasionally getting up once in the night

Getting up once in the night

Getting up between two and four times in the night

Getting up five or more times in the night

15. On average, over the past 4 weeks, how much upset or disruption in your daily activities has the frequency with which you urinate both during the day and the evening caused you?

# No upset or disruption

Very little upset or disruption

Small upset or disruption

Moderate upset or disruption

Severe upset or disruption

16. On average, over the past 4 weeks, how often have you experienced pain or discomfort upon or during urination?

Never

Sometimes

Frequently

Most of the time

All or almost all of the time

## IF NEVER, SKIP TO QUESTION 19

17. On average, over the past 4 weeks, how severe has your pain or discomfort upon or during urination been?

Very mild pain or discomfort

Mild pain or discomfort

Somewhat uncomfortable pain or discomfort

Moderately uncomfortable pain or discomfort

Severe pain or discomfort

18. On average, over the past 4 weeks, how much upset or disruption in your daily activities has your pain upon or during urination caused you?

No upset or disruption

Very little upset or disruption

Small upset or disruption

Moderate upset or disruption

Severe upset or disruption

19. On average, over the past 4 weeks, how often have you had blood in your urine?

Never

# Rarely

# Frequently

Most of the time

All or almost all of the time

**IF NEVER, SKIP TO QUESTION 21**

20. On average, over the last 4 weeks, how much upset or disruption in your daily activities has the blood in your urine caused you?

No upset or disruption

Very little upset or disruption

Small upset or disruption

Moderate upset or disruption

Severe upset or disruption

21. On average, over the past 4 weeks, how often did you leak urine?

Never

Once a day

Twice a day

Three to five times a day

Constantly leak urine

**IF NEVER, SKIP TO QUESTION 24**

22. On average over the past 4 weeks, how many incontinence pads or diapers would you use throughout the course of the day?

None

One

Two

Three to five

Six or more

**IF NONE, SKIP TO QUESTION 24**

23. On average, over the past 4 weeks, how much upset or disruption in your daily activities has the use of incontinence pads or diapers caused you?

No upset or disruption

Very little upset or disruption

Small upset or disruption

Moderate upset or disruption

Severe upset or disruption

**Sexual Functioning**

The following questions deal with your sexual functioning. Some questions are very personal in nature and you may leave them blank if they make you uncomfortable. Your responses will be kept confidential.

Please circle the answer that best describes your situation.

24. On average, over the past 4 weeks, what has been your ability to obtain and maintain an erection?

Very good ability to obtain and maintain an erection

Good ability to obtain and maintain an erection

Moderate ability to obtain and maintain an erection

Poor ability to obtain and maintain an erection

No ability or very poor ability to obtain and maintain an erection

25. On average, during the past 4 weeks, how much upset or disruption in your sexual functioning has your ability to achieve and maintain erections caused you?

# No upset or disruption

Very little upset or disruption

Small upset or disruption

Moderate upset or disruption

# Severe upset or disruption

26. On average, during the past 4 weeks, how would you rate your level of sexual interest?

# Very high level of interest

# High level of interest

# Moderate level of interest

# Low level of interest

# No interest

27. On average, during the past 4 weeks, how much has your level of sexual interest caused upset or disruption to your sexual activities?

No upset or disruption

Very small upset or disruption

Small upset or disruption

Moderate upset or disruption

Severe upset or disruption

28. On average, over the past 4 weeks, how happy and contented were you with your sex life?

# Extremely happy or satisfied

# Somewhat happy or satisfied

# Neither happy or unhappy

Somewhat unhappy or dissatisfied

Not happy or satisfied at all

29. Over the past 4 weeks, which of the following statements **best describes** your level of sexual intercourse?

# Not having sexual intercourse by choice

Not having sexual intercourse due to lack of interest

Not having sexual intercourse due to lack of opportunity

Not having sexual intercourse due to inability to obtain and maintain an erection

Having sexually intercourse to some degree
